# Population density effects on gamete traits and fertilisation dynamics under varying sperm environments in mussels

**DOI:** 10.1002/ece3.11338

**Published:** 2024-05-02

**Authors:** Craig D. H. Sherman, Vincent Careau, Clelia Gasparini, Kim J. Weston, Jonathan P. Evans

**Affiliations:** ^1^ School of Life and Environmental Sciences Deakin University Geelong Victoria Australia; ^2^ Department of Biology University of Ottawa Ottawa Ontario Canada; ^3^ Department of Biology University of Padova Padova Italy; ^4^ Centre for Evolutionary Biology, School of Biological Sciences University of Western Australia Crawley Western Australia Australia

**Keywords:** cryptic female choice, genotype by environment, maternal effect, polyspermy, sexual selection, sperm competition

## Abstract

Gamete traits can vary widely among species, populations and individuals, influencing fertilisation dynamics and overall reproductive fitness. Sexual selection can play an important role in determining the evolution of gamete traits with local environmental conditions determining the strength and direction of sexual selection. Here, we test for signatures of post‐mating selection on gamete traits in relation to population density, and possible interactive effects of population density and sperm concentration on sperm motility and fertilisation rates among natural populations of mussels. Our study shows that males from high‐density populations produce smaller sperm compared with males from low‐density populations, but we detected no effect of population density on egg size. Our results also reveal that females from low‐density populations tended to exhibit lower fertilisation rates across a range of sperm concentrations, although this became less important as sperm concentration increased. Variances in fertilisation success were higher for females than males and the effect of gamete compatibility between males and females increases as sperm concentrations increase. These results suggest that local population density can influence gamete traits and fertilisation dynamics but also highlight the importance of phenotypic plasticity in governing sperm–egg interactions in a highly dynamic selective environment.

## Graphical abstract

Our study not only shows that local population density can influence gamete traits and fertilisation dynamics but also highlights the importance of phenotypic plasticity in governing sperm–egg interactions in a highly dynamic selective environment. Our study highlights that the processes modulating fertilisation success are more complex than previously appreciated. Indeed, our work has important implications for interpretation of past studies on compatibility and intrinsic parental effects which have not taken into consideration the fertilisation environment. Furthermore, our results will change the way researchers in this field think about and study how sexual selection targets gametes.

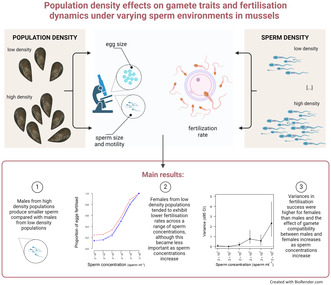

## 1 INTRODUCTION

It is now widely recognised that in most sexually reproducing taxa, sexual selection can continue after mating in the form of sperm competition, where ejaculates from rival males compete for fertilisation (Parker, 1970), and cryptic female choice, where females influence the outcome of these contests (Birkhead & Moller, 1998; Eberhard, 1996; Parker, 2020). This has resulted in a myriad of adaptations in both sexes; for example, selection can favour specific gamete traits that make ejaculates more competitive in the race to fertilise eggs (Lüpold et al., 2020; Pizzari & Parker, 2009; Simmons & Fitzpatrick, 2012), gamete plasticity in response to local environmental conditions (Crean & Marshall, 2008), sperm chemoattractants that function to attract sperm from specific (e.g. compatible) males (Evans et al., 2012; Kekäläinen & Evans, 2017; Lymbery et al., 2017; Oliver & Evans, 2014) or gamete recognition proteins that ultimately determine whether sperm can fuse with an egg (Evans & Sherman, 2013; Levitan & Ferrell, 2006; Palumbi, 1999; Swanson & Vacquier, 2002).

Post‐mating sexual selection has been argued to be particularly important in broadcast spawning organisms that release their gametes into the water for external fertilisation (reviewed in Evans & Lymbery, 2020; Evans & Sherman, 2013; Kekäläinen & Evans, 2018). Indeed, one of the only opportunities for mate choice and mating competition in broadcast spawning organisms occurs through gamete‐level interactions. However, broadcast spawning marine invertebrates face unique challenges that are linked to the spawning environment. In particular, variation in adult population density, and therefore, the density of gametes in the water, is predicted to influence the intensity and direction of post‐mating sexual selection (Franke et al., 2002; Hadlow et al., 2022; Levitan, 2004; Levitan & Ferrell, 2006; Marshall & Bolton, 2007; Parker et al., 1997; Sherman et al., 2015). In low‐density populations, the rapid dilution of gametes can result in low fertilisation rates (Levitan, 2004; Levitan & Petersen, 1995; Styan & Butler, 2000; Vogel et al., 1982), thereby generating selection on gamete traits in both sexes to increase the probablity of egg–sperm encounters and fusion. For example, in females, this may include the production of fewer, larger eggs to increase egg–sperm collision rates, greater investment in sperm‐attracting chemoattractants, greater egg longevity and less selective membrane blocks to sperm penetration (Evans & Lymbery, 2020; Kosman & Levitan, 2014; Levitan, 2006; Levitan & Ferrell, 2006; Marshall & Evans, 2005; Riffell et al., 2004; Yund, 2000). Under these sperm‐limiting conditions, males will maximise their fitness by increasing gamete encounter rates, for example, by producing a smaller number of larger and/or longer‐lived sperm or altering swimming patterns (Benzie & Dixon, 1994; Parker, 1993, 1998; Snook, 2005; Yund, 2000) (but see Crean & Marshall, 2008).

At the other end of the population density spectrum, males from high‐density populations face increased sperm competition, while females face an increased risk of polyspermy—where eggs are fertilised by two or more sperm (usually resulting in cell or zygote death). As a consequence, selection may favour female reproductive strategies that minimise the risk of polyspermy. Specifically, when sperm are abundant and the risks of polyspermy are high, females are predicted to produce smaller eggs, invest less in sperm‐attracting chemoattractants or increase the selectivity of membrane gamete recognition systems to reduce the risk of lethal polyspermy (Kamel et al., 2010; Kosman & Levitan, 2014; Levitan et al., 2007; Levitan & Ferrell, 2006). Males, on the other hand, should produce higher numbers of smaller sperm (Parker, 1998, 2006; Snook, 2005) or release smaller quantities of sperm over a longer period of time (Bode & Marshall, 2007; Marshall & Bolton, 2007).

There are likely to be profound implications for sexual selection associated with variation in sperm availability in broadcast spawners. For example, as population density (and sperm abundance) increases, selection should favour reproductive strategies in males that further exacerbate the reproductive costs incurred by females (polyspermy) (Evans & Lymbery, 2020). This complex interplay between population density and the differential selection pressures faced by males and females can lead to sexual conflict, where the optimal mating strategy of one sex reduces the reproductive fitness of the other (Kamel et al., 2010; Parker, 2006). Furthermore, it has been widely predicted that in broadcast spawners, variation in sperm abundance (e.g. driven by population density but also the inevitable dilution of sperm following spawning) may lead to dramatic departures from the classic ‘Bateman paradigm’ of stronger sexual selection on males compared to females (see Arnold, 1994; Evans & Lymbery, 2020; Levitan, 1998). Briefly, in his famous fruit fly experiments, Bateman (1948) concluded that females are typically constrained by the high costs of gamete production, while males are rarely limited by the capacity to produce sperm. Given these typical patterns, females are expected to exhibit low variance in reproductive fitness (competition is low) while males must compete for available mating opportunities leading to higher variance in reproductive success (see also Davies et al., 2023 for a more recent test of these ideas). However, when sperm become limiting (as is typical in broadcast spawners) or conversely when sperm are over‐abundant, we can expect departures from such patterns, whereby female reproductive success may vary more than males, due either to sperm limitation (many eggs go unfertilised) or sperm over‐abundance (high developmental failure due to polyspermy) (e.g. see Levitan, 2004). Despite these predictions, however, there are only a limited number of studies that have explored selection on gamete traits and fertilisation dynamics from natural populations with varying adult densities (however, see Levitan, 2002, 2004; Levitan & Ferrell, 2006; Levitan, 2012).

Blue mussels of the genus *Mytilus* are external spawners and provide an ideal experimental system for understanding the combined effects of population density, gamete traits and sperm abundance on fertilisation dynamics (Evans et al., 2012; Evans & Lymbery, 2020; Sherman et al., 2015). Blue mussels are found on hard substrates of nearshore and intertidal habitats in temperate and subarctic regions of the northern and southern hemispheres (Hilbish et al., 2000) and are predominant species found in temperate Australian waters (Ab Rahim et al., 2016; Popovic et al., 2020; Westfall & Gardner, 2013; Zbawicka et al., 2021). Populations vary naturally in animal abundance (Cockrell et al., 2015) with likely concomitant variation in sperm concentrations among natural spawning events. Moreover, recent work on Australian *Mytilus* has revealed that ecologically relevant variation in sperm concentrations can have dramatic effects on fertilisation kinetics in terms of exposing compatibility‐based selection, whereby male–female gametic compatibility effects are only observed at high sperm concentrations (Sherman et al., 2015). Furthermore, there is evidence from *Mytilus* for strong patterns of gamete selectivity, such that specific combinations of gametes from both sexes generate predictable differences in fertilisation rates and offspring viability (Evans et al., 2012; Lymbery et al., 2017; Oliver & Evans, 2014; Sherman et al., 2015). However, the extent to which such patterns depend on the fertilisation environment has yet to be determined.

In this study, we explore the effect of adult population density on variation in gamete traits and fertilisation dynamics across a range of sperm concentration environments in mussels from natural populations. We expected that selection would have acted on gamete traits in both sexes to optimise fertilisation success under different egg–sperm ratios. Specifically, we explore whether population density influences gamete traits in both sexes and whether experimental adjustments to sperm–egg ratios during in vitro fertilisation trials influence fertilisation kinetics and pairwise patterns of fertilisation rates (i.e. gamete compatibility). One expectation from the latter trials is that eggs will become more selective as the risk of polyspermy (high sperm concentrations) increases, resulting in stronger gametic (male‐by‐female) interactions under high sperm densities.

## 2 MATERIALS AND METHODS

### 2.1 Collections and spawning of animals

We collected mussel broodstock from high‐ and low‐density populations within each of three locations in Port Phillip Bay, Victoria, Australia, during the May winter spawning season. These three locations included Williamstown (−37.861695°, 144.912751°), Geelong (−38.148770°, 144.390864°) and Portarlington (−38.111790°, 144.659556°). The density of mussels from low‐ and high‐density populations at each location was estimated from six replicate 0.5 m × 0.5 m quadrats and ranged from a mean (±S.E.) of 317.3 (±60.4) to 1154.7 (±139) mussels m^−2^ at Williamstown, 306.7 (±67.8) to 981.3 (±88.3) mussels m^−2^ at Geelong and 274.7 (±42.6) to 962.7 (±72.0) mussels m^−2^ at Portarlington. Mussels were transported to the Victorian Marine Science Consortium research laboratories at Queenscliff and held in flow‐through tanks at ambient temperature (16°C). All animals were cleaned of epiphytes and other debris and used for spawning on the day of collection using the approach of Pettersen et al. (2010).

### 2.2 Collection of gametes

Gametes were harvested by inducing spawning using standard thermal stress protocols (Pettersen et al., 2010). This involved cycling seawater temperature between 16 and 24°C to induce spawning. Males and females were identified at the time of gamete release, rinsed with filtered seawater and isolated into individual spawning chambers (120 × 175 × 70 mm). Rinsing individuals and then conducting spawnings in individual containers would have eliminated the possibility of sperm from non‐focal males fertilising a female's eggs (i.e. avoiding sperm contamination). Eggs were rinsed through a 125 μm mesh, and sperm through a 30 μm mesh, to remove any debris released from the adult mussel during spawning. The concentration of sperm for each male was determined from three replicate counts using an improved Neubauer haemocytometer, and sperm concentrations were initially standardised to 2 × 10^8^ sperm mL^−1^. A serial sperm dilution was then carried out to obtain the desired experimental concentrations of sperm for our experiments: 2 × 10^2^; 2 × 10^3^; 2 × 10^4^; 2 × 10^5^; 2 × 10^6^; and 2 × 10^8^ sperm mL^−1^. Egg concentrations were estimated from three replicate counts using a Beckman multisizer™ 3 Coulter counter and standardised to 2000 eggs mL^−1^ (stock egg solution).

### 2.3 Fertilisation assays

**FIGURE 1:**
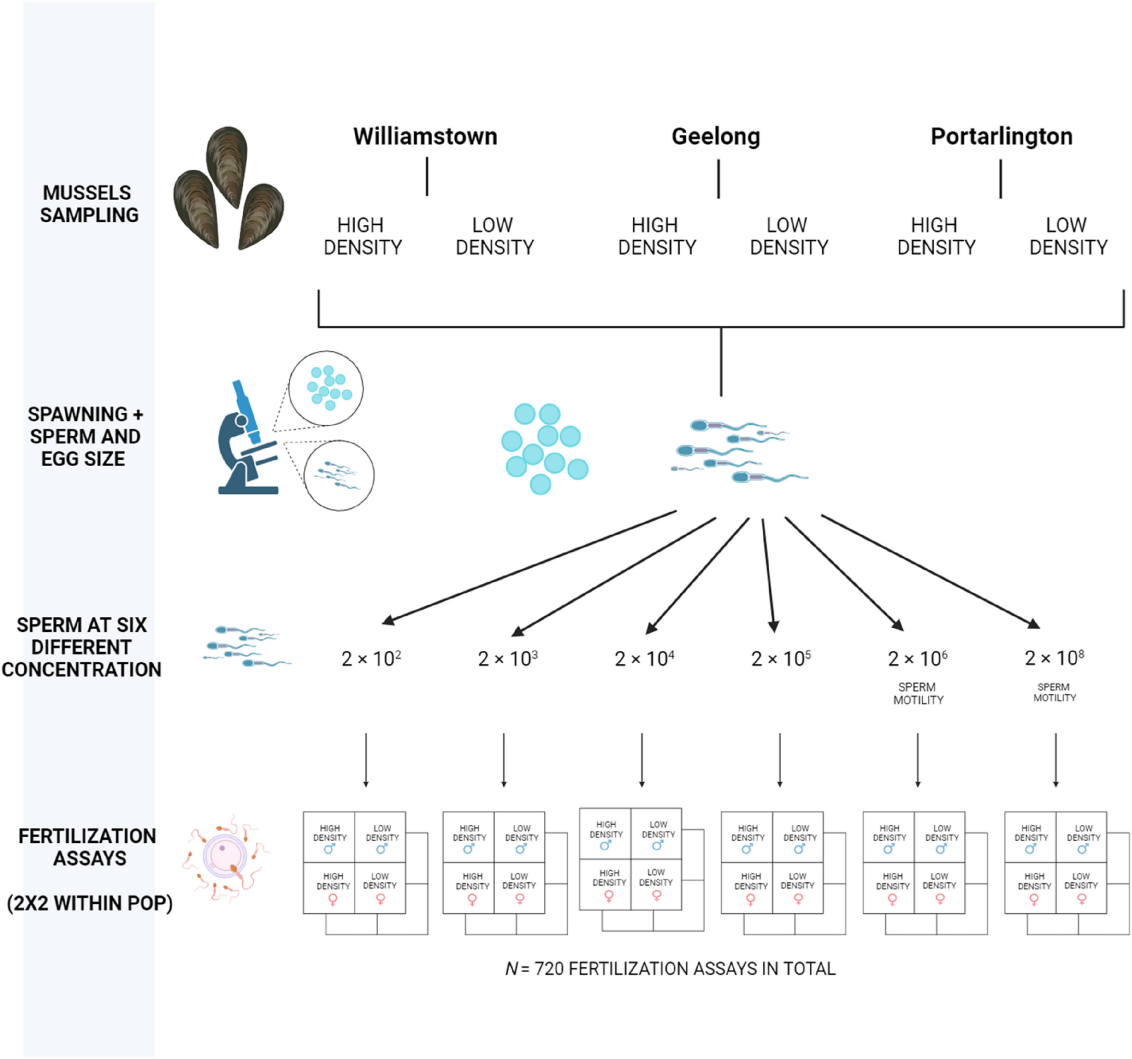
Schematic diagram of the in vitro fertilisation trials with each cross involving two males (one from a high‐density and one from a low‐density population) crossed with two females (one from a high‐ and one from a low‐density population) in every pairwise combination. Created with BioRender.com.

Our block cross‐classified design involved a series of 2 × 2 in vitro fertilisation trials (following the analogous block North Carolina II quantitative genetic breeding design described by Lynch and Walsh (1998)), each involving two males (one from a high‐density and one from a low‐density population from the same location) crossed with two females (one from a high‐ and one from a low‐density population from the same location) in every pairwise combination (Figure 1). For each male–female cross, two replicate fertilisation assays were carried out across each of the six different sperm concentrations, giving a total of 48 fertilisation assays per block. A total of five blocks were carried out for each of the three locations sampled, giving a total of 15 blocks and 720 fertilisation assays across all three locations. We conducted fertilisation assays in sterile 24‐well cell culture plates with a total fertilisation volume of 2 mL. This consisted of 1 mL of the stock egg solution (2000 eggs total), and 1 mL of sperm solution, resulting in a final egg concentration of 1000 eggs ml^−1^ and sperm concentrations of 1 × 10^2^; 1 × 10^3^; 1 × 10^4^; 1 × 10^5^; 1 × 10^6^; and 1 × 10^8^ sperm mL^−1^ (Figure 1). All fertilisation assays within a block were conducted immediately after setting the gamete concentrations and within 1 minute of each other although we also ensured that the time from the start of spawning to fertilisation was recorded and included in the analysis to control for potential gamete ageing effects (‘gamete age’ in the model). Fertilisation assays were left at room temperature for 3 h before samples were fixed with 1% formalin. For each fertilisation assay, a random sub‐sample of approximately 100 eggs was observed under an inverted microscope at 400× magnification and the number of fertilised eggs was distinguished from unfertilised eggs by counting the number of cells that had undergone cell division, or had a clearly visible fertilisation envelope. Unfertilised eggs were identified from the lack of cell division or the absence of a fertilisation envelope (Sherman et al., 2015). Abnormal eggs were identified by signs of irregular cleavage, incomplete blastula development and dissolution of the egg membrane resulting in deformed embryos and/or the breaking apart of the developing cell cluster (Lewis & Galloway, 2009).

### 2.4 Gamete traits and measures of adult body condition

Female egg size was assessed from three replicate counts using a Beckman multisizer™ 3 Coulter counter (mean number of eggs per female = 8371 ± 1140 SE). To estimate sperm size, digital photographs of sperm were taken using a digital camera attached to a microscope (OLYMPUS IX53, 20× objective). Sperm length was measured for each male using the software ImageJ (http://rsb.info.nih.gov/ij). Sperm length was measured in μm as the distance from the tip of the head (excluding the protruding acrosomal cap or ‘beak’) to the end of the tail. Ten sperm were measured for each male, and the average of the 10 measures was used in the analysis.

Sperm motility was assessed using computer‐assisted sperm analysis (CEROS sperm tracker, Hamilton‐Thorne, Beverley USA). Sperm motility was assessed at two sperm concentrations (10^6^ and 10^8^) to confirm that sperm at the highest concentration had been fully activated by the seawater. To assess within‐individual variation, sperm motility was assessed twice for each male at the two higher sperm concentrations (10^6^ and 10^8^). Two replicate 4 μL aliquots of the sperm solution were placed in two separate wells of a 12‐well multi‐test slide and then covered with a coverslip. Sperm motility was therefore assessed in two subsamples for each male at both sperm concentrations. Given the high reported repeatability of sperm motility measures in blue mussels (Fitzpatrick et al., 2012), we recorded the average of the two subsamples for the ensuing analysis, and caution was taken to analyse sperm in a random order with respect to the sperm concentration used in order to avoid conflating treatment effects with order effects. From the CASA analyses, we obtained the percentage of motile sperm and a series of parameters describing velocity and trajectory of motile sperm. These parameters include average path velocity (VAP), straight‐line velocity (VSL), curvilinear velocity (VCL), lateral head displacement (ALH), beat cross frequency (BCF), straightness (STR) and linearity (LIN). Cut‐offs for static cells were the same as those used in previous experiments by Eads et al. (2016). An average of 276.8 ± 37.0 SE sperm tracks were analysed for each male at each concentration.

As body condition may influence fertilisation success (i.e. mussels in better condition may have higher‐quality gametes), we controlled for this potential confounding effect in our analysis. We recorded shell length (mm) and flesh mass (g) from each brood parent and used these to calculate a condition index using the residuals of flesh weight against shell length.

### 2.5 Statistical analysis

Differences in egg size between high‐ and low‐density populations were assessed using a linear model (LM). This included egg size as the response variable, density as a predictor (fixed effect), location (fixed effect) and female condition as a covariate. Similarly, differences in sperm length between low‐ and high‐density populations were assessed using sperm length as the response variable, density as a predictor (fixed effect), location (fixed effect) and male condition as a covariate.

Principal component analysis (PCA) was used to reduce the motility parameters to two PCs with eigenvalues more than 1, explaining, respectively, 60.75% and 23.35% of the variance (total variance explained: 84.10%). PC1 was predominantly loaded by the three velocity measures (VAP, VSL and VCL) and is therefore interpreted as a measure of sperm swimming speed; PC2 was loaded most strongly by STR and LIN and is therefore interpreted as a measure of sperm swimming straightness. These two PCs (PC1 and PC2) were used in all subsequent analyses, and their relationship with the original motility parameters is detailed in Table 1. We analysed differences in sperm motility between low‐ and high‐density populations at the two sperm concentrations (10^6^ and 10^8^, see above) using a general linear mixed model (GLMM) within the lme4 package of R using PC scores (PC1 and PC2) as dependent variables, location, population density and sperm density as fixed factors with a Density × Density interaction, male identity as a random effect and male condition as a covariate. The same model was run to analyse the percentage of motile sperm (arcsine‐transformed before use). Means are presented with their standard errors (SE).

**TABLE 1:** Principal component analysis of sperm motility parameters.

Component	PC1	PC1	PC2	PC2
	*r*	%	*r*	%
VAP	.94	20.78	−.30	5.62
VSL	.98	22.62	.11	0.77
VCL	.87	17.87	−.38	8.89
ALH	.89	18.44	−.20	2.49
BCF	−.49	5.69	.26	4.07
STR	.38	3.40	.90	49.86
LIN	.69	11.21	.68	28.30

*Note*: These parameters include average path velocity (VAP), straight‐line velocity (VSL), curvilinear velocity (VCL), lateral head displacement (ALH), beat cross frequency (BCF), straightness (STR) and linearity (LIN). Correlation coefficient (*r*) and the percentage contribution of each parameter to the principal component score are provided.

We analysed fertilisation success using Markov‐chain Monte‐Carlo generalised linear mixed models in R (package MCMCglmm (Hadfield, 2010)). With this Bayesian mixed‐model approach, we modelled the proportion of normally and abnormally fertilised eggs following a binomial distribution and obtained both an estimate of the components of variance and an estimate of the interval of credibility. All models were run for 1.3 × 10^7^ iterations, with a thinning interval of 100 (i.e. only one iteration from every 100 in the Markov chain was used to estimate the posterior distribution of the parameters to reduce the occurrence of autocorrelation between successive iterations) and a burn‐in of 3 × 10^5^ (i.e. we discarded the first 3 × 10^5^ models of the simulation to avoid issues with autocorrelation).

For the Bayesian models, we included the fixed effects of location (three levels, Geelong, Portarlington and Williamstown), gamete age (time elapsed between spawning and fertilisation), body condition (see above), sperm concentration (fitted both as a linear and quadratic term), density of the male (high vs. low), density of the female (high vs. low) and their interaction with each other and with sperm concentration. The random effects used in the model included block (*V*_block_), identity of male (*V*_male_), identity of female (*V*_female_) and an interaction between male and female IDs (*V*_male×female_). The residual variance (*V*_*e*_) of the model represents the variance between replicates within a given male and female pair.

A necessary step in Bayesian statistical analyses is to set priors before running the models. The term prior refers to the prior distribution of a parameter before the data are analysed. The level of information of the prior can vary from non‐informative to highly informative. When knowledge about the relationship between the variables in the model is low, it is best to run the model with different priors and to check whether these different priors provide different posterior distributions (Hadfield, 2010). We therefore ran the models using inverse Wishart priors (equivalent to an inverse gamma distribution with shape = scale = 0.001; *V* = 1, nu = 0.002), parameter expanded priors (*V* = 1, nu = 1, alpha.mu = 0, alpha.*V* = 1000) and non‐informative improper priors (*V* = 1e‐16, nu = −2). Although we present results from the model using parameter expanded priors, the conclusions did not qualitatively change according to prior specifications. We inspected the 95% highest posterior density (HPD) intervals associated with each fixed effect to check whether they overlapped with zero. A 95% HPD interval contains most of the posterior distribution and is analogous to a confidence interval in the frequentist approach; we considered estimates with 95% HPD intervals overlapping with zero as non‐significant.

We ran an initial model for the proportion of normally fertilised eggs while excluding data from the highest sperm concentration to facilitate the interaction between sperm concentration and population density (i.e. including the highest sperm concentration made the fertilisation curve very complex). To model the proportion of abnormally fertilised eggs, we ran a second model while keeping data in the highest sperm concentration. The proportion of abnormally fertilised eggs was very low at other sperm concentrations.

We ran a third model, this time including data from all sperm concentrations and allowed variance components to vary with sperm concentration. We used the ‘idh()’ function to model heterogeneous variance components for *V*_male_, *V*_female_ and *V*_male×female_ according to each concentration. Hence, there were 20 variance components estimated in this model (i.e. *V*_male_, *V*_female_ and *V*_male×female_ estimated separately for sperm concentrations and a single *V*_block_ and *V*_*e*_). By contrast to Gaussian data, with binomial data, it is not recommended to compare different models using the deviance information criteria (an index produced by MCMCglmm models that balance the fit of the model based on the number of parameters used in the model). Thus, we cannot formally test whether model fit was improved by allowing heterogeneous variance components across sperm concentrations. Instead, we inspected the 95% HPD intervals associated with each random effect to check whether they overlapped. We considered estimates with non‐overlapping 95% HPD intervals as significantly different (Hadfield, 2010). Note that, as the lower limit of a variance component is bound to zero, its lower 95% HPD can be extremely close to, but cannot overlap zero. Thus, inspection of the HPDs cannot be formally used to test whether a variance component is significantly greater than zero (Hadfield, 2010). Nevertheless, the 95% credible intervals around the variance estimates provide a measure of the precision of the estimate and allowed us to test whether *V*_male×female_ differed across sperm concentrations.

## 3 RESULTS

### 3.1 Gamete traits

**FIGURE 2:**
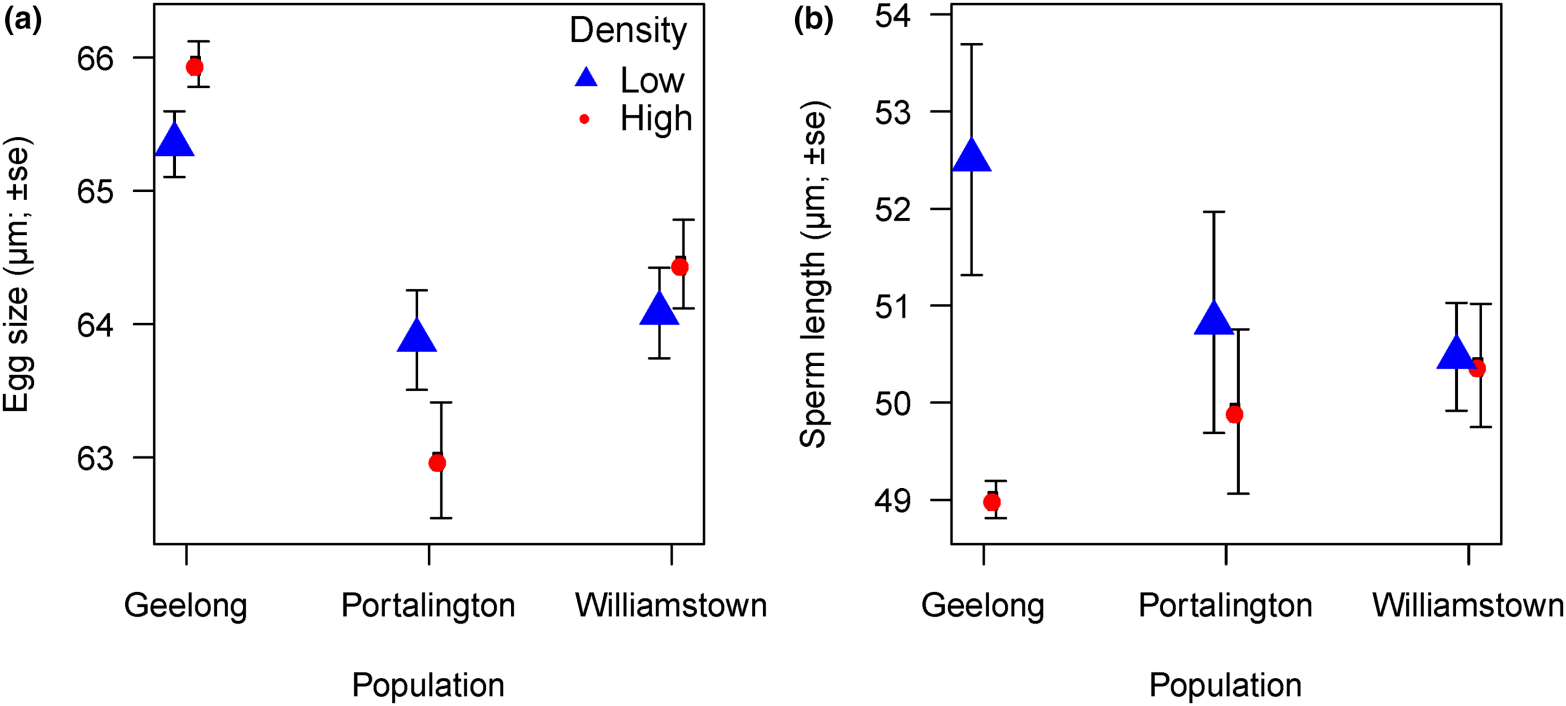
Gametes traits (egg size and sperm length) of Australian blue mussels obtained from individuals from high‐ and low‐density populations from three locations.

Egg size did not differ significantly between females from high‐ and low‐density populations (mean egg size: Female_(high)_ = 64.36 μm ± 0.359 vs. Female_(low)_ = 64.35 μm ± 0.244, LM: *F*_1,25_ = 0.002, *p* = .962). However, we did detect a significant difference in egg size among the three locations (mean egg size: Williamstown = 64.27 μm; ±0.233, Geelong = 65.65 μm ± 0.179, Portarlington = 63.43 μm ± 0.308 and LM: *F*_2,25_ = 14.42, *p* < .0001). The ‘density by location’ interaction was marginally non‐significant (*F*_2,23_ = 3.31, *p* = .055), indicating minor egg size differences at high versus low densities from one location to another (Figure 2a). There is little variance in sperm length within an ejaculate with the coefficient of variation ranging from 1.9 to 11.8 (mean ± SE = 4.06 ± 0.421). Our analysis of sperm length revealed a significant difference between high‐ and low‐density populations (LM: *F*_1,25_ = 4.39, *p* = .046), with sperm from low‐density populations being longer compared with sperm from high‐density populations (mean sperm length: low density = 51.13 μm ± 0.547 cf. high density = 49.86 μm ± 0.387). There was no significant difference in sperm length among locations (LM: *F*_2,25_ = 0.027, *p* = .974). The ‘density by location’ interaction was marginally significant (*F*_2,23_ = 3.48, *p* = .048), indicating minor sperm length differences at high versus low densities from one location to another (Figure 2b).

There was no effect of population density (*F*_1,22.029_ = 0.3519, *p* = .5591) or sperm density (*F*_1,22.299_ = 0.2635, *p* = .6128) on sperm motility PC1 scores, or their interaction (*F*_1,22.7_ = 0.05, *p* = .8253). Similarly, PC2 scores were not affected by population density (*F*_1,271_ = 0.25, *p* = .618), sperm density (*F*_1,29.01_ = 0.16, *p* = .689) or their interactions (*F*_1,29.3_ = 0.06, *p* = .811). Male condition also did not affect sperm motility PC1 (*F*_1,35.0_ = 0.013, *p* = .911) or PC2 (*F*_1,41.3_ = 0.36, *p* = .550) scores. The percentage of motile sperm was not affected by population density (*F*_1,26.9_ = 0.05, *p* = .819), but it was affected by sperm density (*F*_1,26.2_ = 46.7, *p* < .001). We also detected no significant effects of the interaction between population and sperm density (*F*_1,26.7_ = 0.020, *p* = .890) or the effect of male condition (*F*_1,36.0_ = 1.89, *p* = .178) on the percentage of motile sperm. Sperm were on average more motile at the 1 × 10^8^ concentration (31.82% ± 2.22) compared to the 1 × 10^6^ concentration (14.42% ± 2.85).

### 3.2 Fertilisation

**FIGURE 3:**
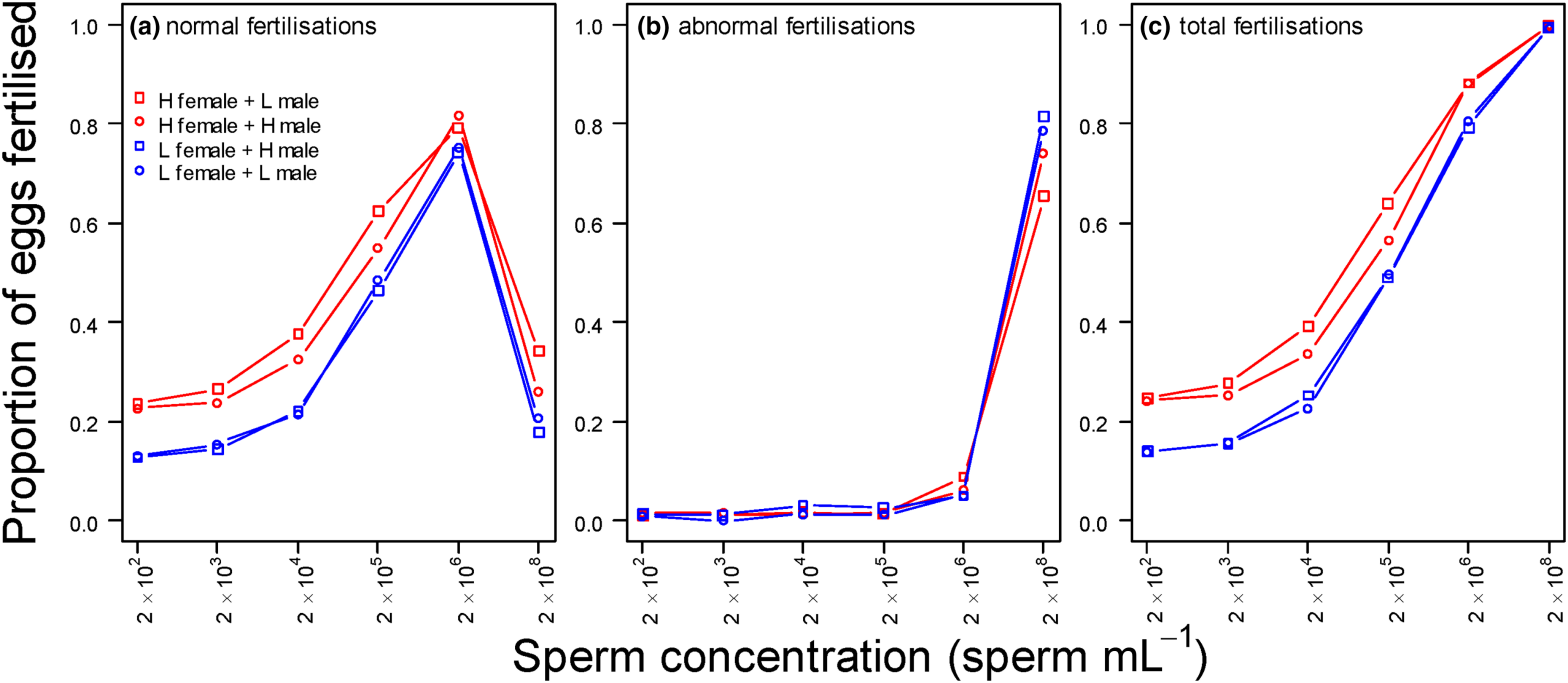
Proportion of (a) normally, (b) abnormally and (c) total (=normally + abnormally) fertilised eggs as function of sperm concentration in mussels collected from high (H) and low (L) population density and mated in a fully factorial fertilisation design. For each experimental block, four females were paired with four males (and vice versa) at six different sperm concentrations. Each line within a panel represents the mean of a specific male and female population density. Hence, comparing the red versus blue lines shows the difference between H and L females. Comparing squared versus circles shows the differences between H and L males.

The percentage of successfully fertilised eggs steeply increased with sperm concentration but dropped significantly beyond 1 × 10^6^ (Figure 3a). Beyond this concentration, ~60 to 80% of eggs were abnormally fertilised (Figure 3b). Therefore, had we simply counted fertilised versus unfertilised eggs, without making a distinction between normally versus abnormally fertilised eggs, we would have seen a continuous increase in proportion of fertilised eggs (Figure 3c) while in fact many of the fertilised eggs at the highest sperm concentration (1 × 10^8^) were non‐viable.

In the first Bayesian model, we analysed the proportion of normally fertilised eggs after excluding the highest sperm concentration (1 × 10^8^). There was a significant effect of sperm concentration (both linear and quadratic terms), female density and a significant interaction between female density and sperm concentration (Table 2). Females from low‐density areas had lower fertilisation success across all sperm concentrations (Figure 1a), but the significant interaction indicated that this effect becomes less important as sperm concentration increases (Table 2). We detected no effect of male density on fertilisation success (Table 2). In the second model, in which we analysed the proportion of abnormally fertilised eggs at the highest sperm concentration (1 × 10^8^), we found no significant effect of location, body condition, gamete age, female density and male density (or their interaction) on the proportion of abnormally fertilised eggs (Table 3).

**TABLE 2:** Parameters from a mixed model of proportion of eggs normally fertilised across different sperm concentrations in mussels, fitted using a Bayesian approach.

	Estimate	95% HPD interval	
		Lower	Upper
(a) Random effects:			
*V*_block_	0.706	0.000	2.223
*V*_male_	0.058	0.000	0.184
*V*_female_	1.416	0.575	2.500
*V*_male×female_	0.045	0.000	0.127
*V*_*e*_	1.430	1.224	1.630
(b) Fixed effects:			
Intercept	0.443	−2.076	3.276
Location_[Portarlington]_	−0.702	−2.262	1.056
Location_[Williamstown]_	−1.411	−3.393	0.345
Male body condition index	−0.255	−0.508	−0.046
Female body condition index	−0.203	−0.894	0.637
Male gamete age	0.001	−0.008	0.010
Female gamete age	0.000	−0.011	0.011
Sperm concentration	33.126	28.735	37.249
Sperm concentration^2^	13.124	10.690	15.503
Male density_[low]_	0.113	−0.442	0.671
Female density_[low]_	−1.308	−2.266	−0.225
Male_[low]_ × female_[low]_ density	−0.106	−0.551	0.375
Male density_[high]_ × sperm concentration	−0.027	−0.178	0.113
Female density_[low]_ × sperm concentration	0.195	0.062	0.341

*Note*: Shown are posterior means and the 95% highest posterior density (HPD) intervals for (*a*) random effects of measurement block (*V*_block_), male identity (*V*_male_), female identity (*V*_female_), specific combinations of males and females (*V*_male×female_) and specific environment (*V*_*e*_; residual variance) and (*b*) fixed effects of location (three levels; Geelong as the reference), body condition, gamete age (minutes), sperm concentration (continuous variable fitted both as linear and quadratic term), density of the male, density of the female and their interactions with each other and with sperm concentration.

**TABLE 3:** Parameters from a mixed model of proportion of eggs abnormally fertilised at the highest sperm concentration in mussels, fitted using a Bayesian approach.

	Estimate	95% HPD interval	
		Lower	Upper
(a) Random effects:			
*V*_block_	3.928	0.001	11.090
*V*_male_	0.834	0.000	3.418
*V*_female_	1.788	0.000	5.956
*V*_male×female_	4.100	1.136	7.083
*V*_*e*_	0.113	0.031	0.203
(b) Fixed effects:			
Intercept	2.762	−3.245	7.958
Location_[Portarlington]_	−1.504	−4.822	2.203
Location_[Williamstown]_	−3.039	−6.818	1.163
Male body condition index	0.218	−0.690	1.312
Female body condition index	0.460	−0.827	1.963
Male gamete age	0.015	−0.008	0.035
Female gamete age	−0.010	−0.029	0.011
Male density_[low]_	−0.606	−2.470	1.123
Female density_[low]_	0.725	−1.007	2.613
Male_[low]_ × female_[low]_ density	0.618	−1.378	2.896

*Note*: Shown are posterior means and the 95% highest posterior density (HPD) intervals for (*a*) random effects of measurement block (*V*_block_), male identity (*V*_male_), female identity (*V*_female_), specific combinations of males and females (*V*_male×female_) and specific environment (*V*_*e*_; residual variance) and (*b*) fixed effects of location (three levels; Geelong as the reference), body condition, gamete age (minutes), density of the male, density of the female and their interaction.

**FIGURE 4:**
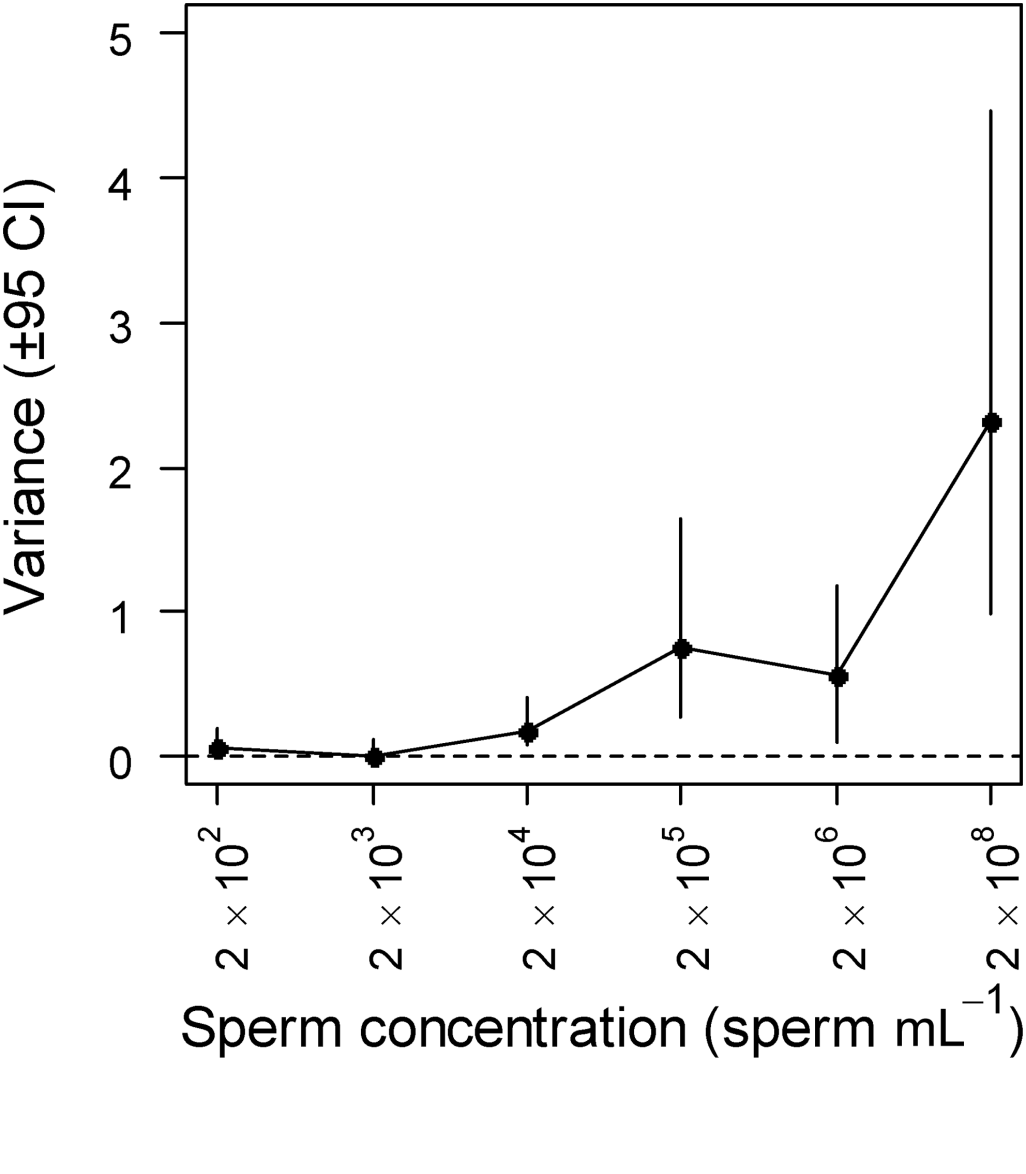
Variance in fertilisation success attributed to the interaction between male and female identity (*V*_male×female_) across sperm concentrations in mussels. Black dots show posterior modes and the lines show 95% confidence intervals (CI; highest posterior density intervals) from the MCMCglmm model.

Finally, our third Bayesian model revealed some differences in the variance estimates across sperm concentrations. The variance estimates associated with intrinsic male effects (*V*_male_) were low across all sperm concentrations (range: <0.01–0.01; Table 4). By contrast, effects attributable to females (*V*_female_) were relatively high (range: 0.62–4.96; Table 4). The ‘male × female’ interaction effects (*V*_male×female_) were relatively low at low sperm concentration (Figure 4) but increased at higher sperm concentration (Table 4, Figure 4).

**TABLE 4:** Variance estimates from a mixed model of proportion of eggs normally fertilised across different sperm concentrations in mussels, fitted using a Bayesian approach.

Variance component	Posterior mode	95% HPD	
		Lower	Upper
*V*_block_	0.28	<0.01	1.82
*V*_male_ [2 × 10^2^]	<0.01	<0.01	0.05
*V*_male_ [2 × 10^3^]	<0.01	<0.01	0.03
*V*_male_ [2 × 10^4^]	<0.01	<0.01	0.08
*V*_male_ [2 × 10^5^]	0.01	<0.01	2.19
*V*_male_ [2 × 10^6^]	0.01	0.00	1.25
*V*_male_ [2 × 10^8^]	0.02	0.00	2.71
*V*_female_ [2 × 10^2^]	1.51	0.68	2.68
*V*_female_ [2 × 10^3^]	1.12	0.56	2.18
*V*_female_ [2 × 10^4^]	0.62	0.23	1.49
*V*_female_ [2 × 10^5^]	1.96	0.00	4.97
*V*_female_ [2 × 10^6^]	4.67	1.28	16.19
*V*_female_ [2 × 10^8^]	4.96	1.16	41.24
*V*_male×female_ [2 × 10^2^]	0.06	<0.01	0.19
*V*_male×female_ [2 × 10^3^]	<0.01	<0.01	0.12
*V*_male×female_ [2 × 10^4^]	0.18	0.09	0.42
*V*_male×female_ [2 × 10^5^]	0.75	0.27	1.65
*V*_male×female_ [2 × 10^6^]	0.56	0.11	1.17
*V*_male×female_ [2 × 10^8^]	2.33	0.99	4.47
*V*_*e*_	0.05	0.03	0.07

*Note*: Shown are posterior modes and the 95% highest posterior density (HPD) intervals for random effects of measurement block (*V*_block_), male identity (*V*_male_), female identity (*V*_female_), specific combinations of males and females (*V*_male×female_) and specific environment (*V*_*e*_; residual variance). *V*_male_, *V*_female_ and *V*_male×female_ were fitted heterogeneously for each sperm concentration.

## 4 DISCUSSION

In this study, we tested for signatures of post‐mating selection on gamete traits in relation to population density, and possible interactive effects of population density and sperm concentration on sperm motility and fertilisation rates. Our study yielded four key results: (1) males from high‐density populations produce smaller sperm compared with males from low‐density populations, although we found no difference in egg size between females from different population densities; (2) females from low‐density populations have lower fertilisation success, although this becomes less important as sperm concentration increases; (3) variances in fertilisation success were higher for females than males; and (4) gamete compatibility between males and females increases as sperm concentrations increase.

We found that males from high‐density populations, where sperm competition would be expected to be more prevalent (Evans & Lymbery, 2020), produced smaller sperm compared to males from low‐density populations. To the extent that sperm production trades‐off against sperm size (Gomendio et al., 1991; Parker, 1982; Tourmente et al., 2011), this result supports the prediction that an increase in the overall magnitude of sperm competition should select for a greater number of smaller sperm, while lower sperm competition risk should result in the production of fewer longer sperm (Gage & Morrow, 2003; García‐González & Simmons, 2007; Lüpold et al., 2020; Parker, 1982; Stockley et al., 1997). These results support comparative studies of fish that show sperm length decreased with sperm competition risk (Stockley et al., 1997), although some studies have found the opposite, with among‐species comparisons showing a positive relationship between sperm competition risk and sperm size (Byrne et al., 2003; Gage, 1994; Johnson et al., 2013; Lifjeld et al., 2010). The reason for such varying and contrasting results likely arises from the complex relationship between sperm morphology and sperm performance (e.g. swimming speed and/or fertilisation potential). In some species (especially internal fertilisation taxa), longer sperm may be associated with improved swimming performance and provide a competitive advantage under sperm competition (Gomendio et al., 1991; Johnson et al., 2013; Parker et al., 2010). However, sperm length may also be associated with sperm longevity, with longer, larger sperm living longer than smaller sperm (Helfenstein et al., 2008). If so, producing longer‐lived sperm may confer an important advantage in broadcast spawners in low‐density populations, allowing more time for sperm–egg encounters and increased fertilisation success (however, see Levitan, 2000). A study of the broadcast spawning tubeworm *Galeolaria gemineoa* showed that sperm with small heads but long tails were favoured in high‐concentration environments, whereas sperm with long heads were favoured at low concentrations and old ages (Johnson et al., 2013). The longer sperm produced by males from low‐density populations detected in this study may provide an advantage if they have greater longevity, although longevity was not directly assessed in this study. Our analysis also showed that longer sperm from low‐density populations did not show greater motility or swimming speeds, suggesting that the larger size did not confer a swimming advantage. The relation between sperm size and fertilisation success under sperm competition is complex and appears to be highly context dependent and likely to covary with sperm swimming traits and longevity. Future studies that quantify the relationship among sperm size, longevity and motility in the context of sperm competition risk are needed to elucidate their effects on fertilisation success and understand how sexual selection may be shaping these reproductive traits.

Theoretical and empirical studies predict that egg size should reflect adult density, with females from low‐density populations producing larger eggs than their high‐density counterparts, because larger eggs present larger targets for searching sperm (Crean & Marshall, 2008; Evans & Lymbery, 2020; Levitan, 2006). In the present study, we found no difference in mean egg size between females from high‐ and low‐density populations. However, egg size is not the only mechanism available to females for increasing the effective target size of their eggs. For example, it is well established that the eggs of many broadcast spawners release sperm chemoattractants, which are thought to increase the effective target size of eggs, thus making them more ‘visible’ to searching sperm (for theory, see Jantzen, 2001). While we currently lack explicit evidence that females can facultatively adjust the production of sperm chemoattractants to match the fertilisation environment, or that selection may favour increased production of such attractants in populations with persistently low adult densities (and therefore sperm limited), the idea of testing for differences either in the composition or volume of sperm chemoattractants across populations has merit. Such an adaptation may represent a more cost‐effective mechanism for increasing the target size of eggs compared to increasing the structural size of eggs in sperm‐limited environments and offer an exciting area for future research.

We assessed fertilisation rates of females from low‐ and high‐density populations across a range of sperm concentrations. As population densities decrease and the risk of sperm limitation increases, we expected that females would produce eggs that are more readily fertilised (i.e. greater fertilisation success at lower sperm concentrations), while in high sperm density environments, females should increase ovum defences to reduce the risk of polyspermy (Firman & Simmons, 2013; Frank, 2000; Kosman & Levitan, 2014). Surprisingly, we found no evidence to support these ideas. Indeed, we found that females from high‐density populations produced eggs that were more readily fertilised at lower sperm concentrations than eggs from females originating from low‐density populations, although this difference became progressively less apparent as sperm concentrations increased. The underlying reasons and possible adaptive basis for these patterns, which appear to be counterintuitive, are unclear to us and we require further study to understand the possible fitness implications of these findings.

Interestingly, we found not only that the variance components for (normal) fertilisation rates across the range of sperm concentrations were generally higher in females than in males but also that variances for females were initially high at lower sperm concentrations (2 × 10^2^ & 2 × 10^3^ mL^−1^), decreased when sperm concentrations were at an intermediate level (2 × 10^4^ & 2 × 10^5^ mL^−1^), before rising sharply when sperm concentrations exceeded 2 × 10^6^ mL^−1^. These findings support the accumulating theory and evidence from broadcast spawning invertebrates (reviewed in Evans & Lymbery, 2020) and other systems with similar reproductive systems (e.g. wind‐pollinated plants; Tonnabel et al., 2019) that patterns of sexual selection can differ radically from the typical ‘Bateman paradigm’ of higher variance in reproductive success in males than females. Indeed, qualitatively similar patterns of reproductive variance have been reported in other broadcast spawning invertebrates (Levitan, 2004), suggesting that under sperm limitation, where average fertilisation rates are low, there will be heightened opportunity for selection on female traits that improve fertilisation rates. Under such conditions, for example, we might expect stronger selection for increased egg size and/or the heightened production of chemoattractants that increase sperm–egg encounter rates (see Evans & Lymbery, 2020). Under intermediate sperm concentrations, fertilisation rates were generally high (~80%) and the variance in normal fertilisation rates was consequently very low. By contrast, despite high overall fertilisation rates in the highest sperm concentration groups (>2 × 10^6^ mL^−1^), the proportion of normally fertilised eggs decreased dramatically at high sperm concentrations with a concomitant increase in the variance in normally fertilised eggs (see Figure 1a). Together these findings suggest that the opportunity for selection on egg traits will depend critically on local spawning conditions and that such patterns may be reflected on a broader spatial scale in divergent populations with varying adult densities.

Our fertilisation assays indicated that as sperm concentration increased, gametic compatibility effects became increasingly important. This suggests that under sperm‐limited conditions, where the risk of fertilisation failure is higher, any benefits associated with selecting genetically compatible sperm (Kosman & Levitan, 2014; Oliver & Evans, 2014) are offset by the direct costs of leaving many eggs unfertilised. However, as sperm concentration increases, eggs can afford to become ‘choosier’ as this will ensure that fertilisations are biased towards genetically compatible sperm (Lymbery et al., 2017; Sherman et al., 2015) while avoiding the direct costs associated with polyspermy. Although we have yet to understand the mechanistic basis that underlies these dynamic patterns of sperm–egg interaction, we suspect that sperm chemoattraction and gamete surface proteins play important roles in differentially regulating sperm–egg encounter rates across the sperm concentration continuum (Evans & Sherman, 2013). Irrespective of the underlying mechanisms, our results provide further support to an increasing number of studies that have shown that local environmental conditions can influence the magnitude of compatibility effects between male–female combinations (Levitan et al., 2007; Levitan & Ferrell, 2006; Lymbery & Evans, 2013; Nystrand et al., 2011; Rudin‐Bitterli et al., 2018; Sherman et al., 2015). Collectively, these studies highlight the importance of testing for compatibility effects across a range of ecologically relevant environmental conditions.

In conclusion, our study revealed significant effects of population density and sperm concentration on gamete morphology and fertilisation dynamics respectively. While the extent of variation at the population level may be determined by both environmental and/or genetic factors, the critical importance of gamete‐level natural and sexual selection in broadcast spawners (Evans & Lymbery, 2020; Evans & Sherman, 2013) leads us to predict that much of the variation we observe in these populations is adaptive. However, we also report highly dynamic patterns of fertilisation across experimentally altered sperm environments, highlighting the importance of phenotypic plasticity in governing sperm–egg interactions and the likely dynamic selective environment in which fertilisation plays out. We eagerly anticipate future work that seeks to understand the extent to which the mechanisms underlying these dynamic patterns of sperm–egg interaction are themselves plastic in their expression.

## AUTHOR CONTRIBUTIONS

**Craig D. H. Sherman:** Conceptualization (lead); data curation (equal); investigation (equal); methodology (equal); project administration (equal). **Vincent Careau:** Conceptualization (equal); data curation (equal); formal analysis (equal); investigation (equal); methodology (equal). **Clelia Gasparini:** Data curation (equal); formal analysis (equal); methodology (equal). **Kim J. Weston:** Data curation (equal); methodology (equal). **Jonathan P. Evans:** Conceptualization (equal); data curation (equal); formal analysis (equal); investigation (equal); methodology (equal); project administration (equal).

## CONFLICT OF INTEREST STATEMENT

The authors declare no conflict of interest.

## Data Availability

Data and associated r‐code for the fertilisation analysis can be found here: https://datadryad.org/stash/share/Yx‐wxEJsD‐SjrHkHG9lVShv1Qw‐KFTAT55vBMvUBVQ4.
